# An Improved Model for Biogenic Ammonium Urate

**DOI:** 10.1021/acs.cgd.3c00789

**Published:** 2023-08-16

**Authors:** Alyssa M. Thornton, Timothy G. Fawcett, James A. Kaduk, YuJai Lin, Jennifer A. Swift

**Affiliations:** † Department of Chemistry, Georgetown University, Washington, District of Columbia 20057, United States; ‡ International Centre for Diffraction Data, Newtown Square, Pennsylvania 19073, United States; § Illinois Institute of Technology, 3101 S. Dearborn Street, Chicago, Illinois 60616, United States; ∥ North Central College, 131 S. Loomis Street, Naperville, Illinois 60540, United States

## Abstract

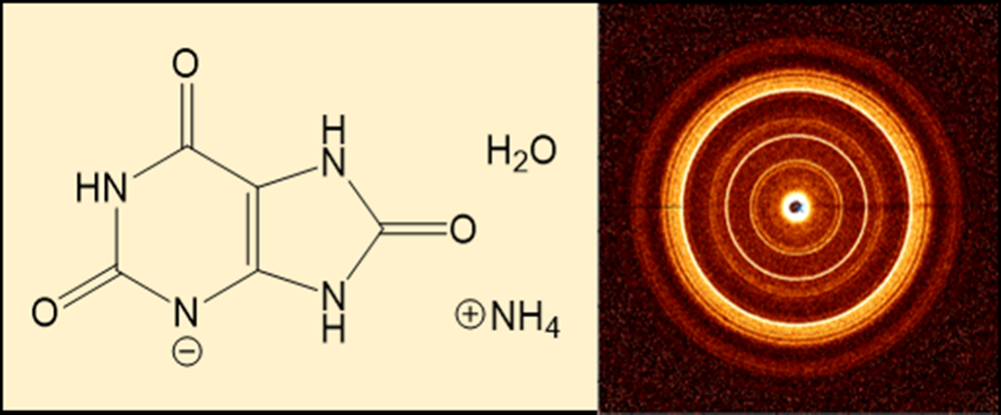

The pathological crystallization of ammonium urate inside the urinary tract is a well-documented medical condition; however, structural studies of the biogenic material have proven challenging owing to its propensity to precipitate as a powder and to exhibit diffraction patterns with widely varying intensities. Using block Rietveld refinement methods of powder diffraction data, here we identify ammonium urate hydrate (AUH) as a likely component in natural uroliths. AUH has a planar 2-D hydrogen-bonded organic framework of urate ions separated by ammonium ions with water molecules residing in bisecting channels. AUH is stable up to 150 °C for short time periods but begins to decompose with prolonged heating times and/or at higher temperatures. Changes in the solid-state structure and composition of synthetic material over a temperature range from 25 to 300 °C are elucidated through thermogravimetric and spectroscopic data, combustion analysis, and time-resolved synchrotron powder X-ray diffraction studies. We contend that biogenic ammonium urate is more accurately modeled as a mixture of AUH and anhydrous ammonium urate, in ratios that can vary depending on the growth environment. The similar but not identical diffraction patterns of these two forms likely account for much of the variability seen in natural ammonium urate samples.

## Abstract 2

Ammonium urate kidney stones are common in some animal species. Here, ammonium urate hydrate (AUH) is identified as a likely component in these pathological deposits. Different ratios of hydrate and anhydrate crystal forms likely account for the variability seen in the powder X-ray diffraction patterns of biogenic samples.

## Introduction

Urolithiasis, the formation of renal calculi, is a common medical condition that affects humans as well as other animals.^1,2^ Such physiologic deposits can have a range of chemical compositions, the frequencies of which vary across different species. Though infrequently identified in humans,^3^ ammonium urate stones are quite common in some canine breeds (e.g., Dalmatians),^4,5^ felines,^6^ and managed bottlenose dolphins.^7−9^ Reptiles are also not immune from developing pathogenic ammonium urate deposits^10,11^ even though some routinely excrete excess nitrogen in that solid form.^12^

Crystallographic data on biogenic ammonium urate are limited, in part because the crystals are poorly formed and rarely pure. Obtaining large synthetic ammonium urate single crystals is also difficult, such that most previous studies have relied on powder X-ray diffraction (PXRD) data. Tettenhorst and Gerkin^13^ were the first to report a unit cell for synthetic ammonium urate (AU) from PXRD data. They considered the possibility that the material could be a hydrate but concluded it was anhydrous based on multiple analytical tests. More recently, Rimer et al.^14,15^ reported a monoclinic structure for synthetic AU determined from both 3D transmission electron microscopy and Rietveld refinement of PXRD data (refcode: TIGZUI). Structures determined by each method were in excellent agreement with one another and with the Tettenhorst and Gerkin^13^ PXRD pattern.

Working with PXRD data from a human urolith and Rietveld refinement methods, Friedel et al.^16^ proposed a different structure with an ammonium urate ammonia composition (refcode: HOZSUL) where the second ammonia site was partially occupied. Notably, their cell volume per urate was about 5% larger than that reported for AU (Table S1). The authors were confident that the unit cell was triclinic, not monoclinic, and noted some line broadening in the patterns which they hypothesized could be related to structural gradients in the material. The data refined to a low *R*_wp_ but had some unusual features including a non-planar urate in a tautomeric form different than in other urate salts.^17−20^ The presence of an ammonia molecule was also not apparent in the elemental analysis, though it was purposefully included in the structure model to align with the measured density of the synthetic material.^13^

In our own studies of ammonium urate, syntheses repeatedly produced materials with a triclinic unit cell matching the one reported by Friedel et al.^16^ but with a composition more accurately described as ammonium urate hydrate (AUH). Based on multiphase Rietveld refinement methods, thermogravimetric and elemental analysis, and time-resolved synchrotron powder diffraction techniques, here we report an improved model for biogenic ammonium urate uroliths. The coexistence of hydrate and anhydrate forms in different ratios helps to explain some of the variability in the X-ray diffraction patterns of different biogenic ammonium urate samples.

## Results and Discussion

### AUH Structure Determination and Properties

AUH was synthesized by adding anhydrous uric acid powder to an ammonium hydroxide solution (excess ammonia) at room temperature for a minimum of 24 h. These simple reaction conditions typically yielded AUH in a phase mixture with smaller amounts of amorphous material and the anhydrous AU form previously reported. With mixed phase samples and small crystallite sizes, our structure solution for AUH followed a Rietveld block refinement strategy. First, an initial energy minimized structure was calculated using VASP^21^ and density functional theory (DFT) methods using edited fragments from published structures. Unit cells, scale factors, and an overall temperature factor were refined, the results were analyzed, and then peak profiles were refined in later cycles. Atomic parameters and site occupancies were selectively refined in the final steps. This approach was applied to PXRD data sets from four different synthetic batches. Water occupancies varied but were close to 0.73 in the three data sets with the highest intensity counts. All successfully refined to the same AUH structure (Table S2, Figure S2) with a final *R*_wp_ between 3.5 - 8.5%, good cell precision, and low residual electron density (*P* – 1: *a* = 3.686 Å, *b* = 10.073 Å, *c* = 10.637 Å, α = 113.46°, β = 90.52°, γ = 91.65°, *V* = 362.07 Å^3^, *Z* = 2).

**Figure 1:**
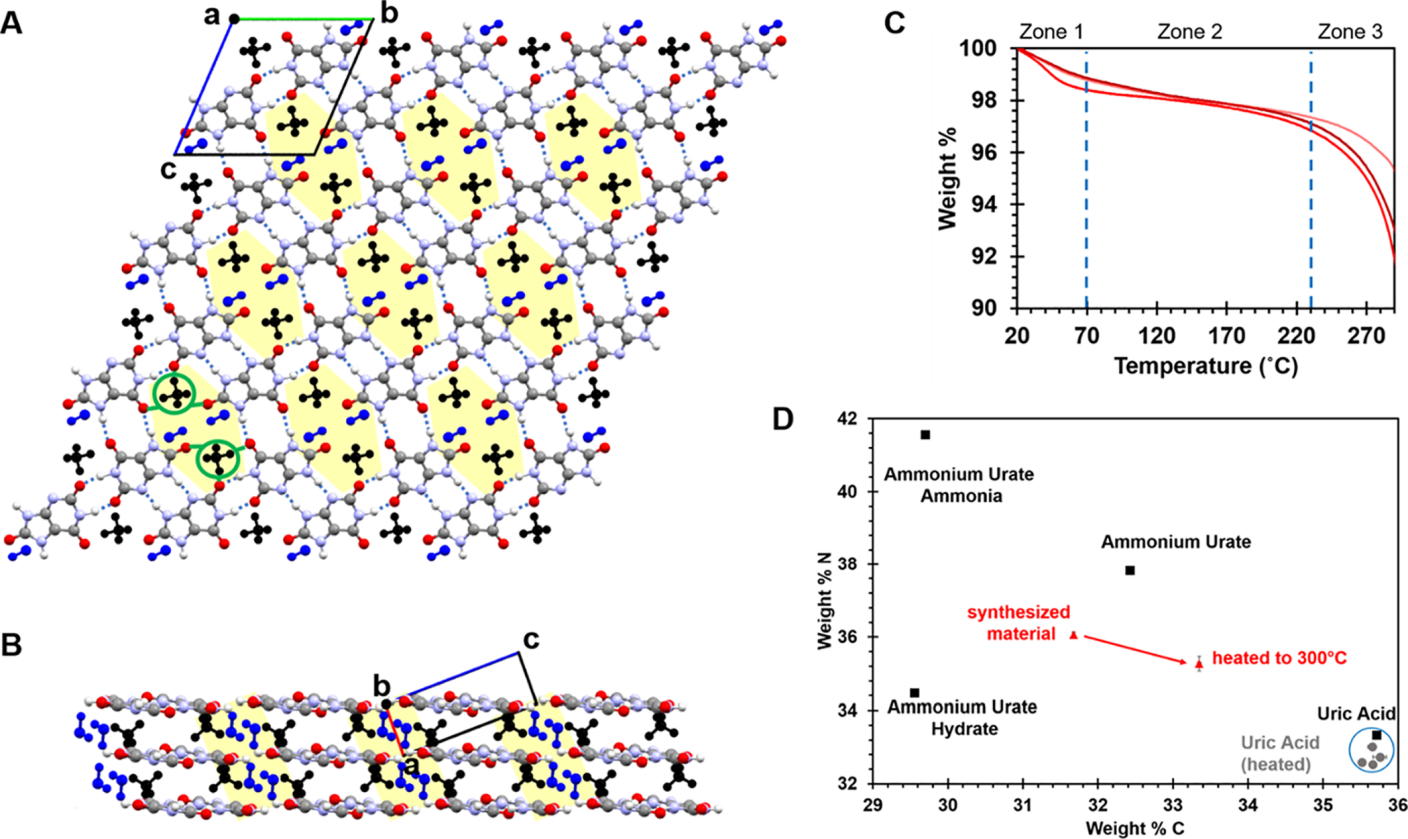
(A, B) Packing diagram of AUH viewed down the *a*-axis and *b*-axis, respectively. Ammonium ions are black and water molecules are blue for clarity. Green circles indicate ammonium ions occupy positions above and below the 2-D urate sheets. (C) TGA thermograms of synthesized material heated at 10 ° C/min. (D) N/C ratios of theoretical compositions (black), and elemental analyses of the synthetic material before and after heating (red), and uric acid heated to the same temperatures (gray).

The AUH structure (Figure 1A,B) consists of robust planar 2-D sheets of urate ions in the (10–1) plane. Urate ions within each plane are bonded side...side in the [011] direction through N_3_...H–N_9_ and O_6_...H–N_10_ (2.69 and 2.88 Å), and head...head along [01–1] through N_1_–H...O_2_ (2.757 Å). Adjacent 2-D urate sheets π-stack along the *a*-axis with an interplanar separation distance of ∼3.5 Å. This assembly creates one-dimensional pores which are partially occupied with water molecules. Ammonium ions reside in between the sheets near the edges of the pores, each forming hydrogen bonds to three urate ions through N–H...O_8_, N–H...O_2_, and N–H...O_6_ (2.55, 2.72, and 2.82 Å, respectively).

The synthesized material was characterized with a combination of thermal, spectroscopic, and elemental analysis methods. Differential scanning calorimetry (DSC) showed two broad endotherms when heated at 10 °C/min to 400 °C (Figure S3). The first transition has an almost immediate *T*_onset_ and a *T*_max_ 94 ± 6 °C, while the second has a *T*_onset_ ∼ 280 °C and a *T*_max_ 321 ± 2 °C. When the material was exposed to ambient air for 4 days before DSC analysis, the first transition was absent. This suggests that the first endothermic event, when observed, corresponds to surface water loss. TGA thermograms (Figure 1C) were highly reproducible, showing a 1.2 ± 0.3% loss between 23−70 °C (zone 1) and another 1.7 ± 0.2% loss between 70−230 °C (zone 2). The much larger weight loss and higher temperature endotherm were assumed to correspond to decomposition.

Water and ammonia are very similar in size, molecular weight, and electron density, such that neither the PXRD data nor the thermal analyses can unambiguously distinguish between the two. However, elemental analyses inform on this point. Figure 1D compares the N/C ratios for theoretical compositions (black) and measured values of synthetic material (red) before and after heating to 300 °C under nitrogen. Theoretical values for AUH and ammonium urate ammonia^16^ assume full occupancy for water and ammonia, respectively. The synthesized material is a mixture, with a N/C ratio falling in between that of AUH and AU rather than between ammonium urate ammonia, and AU. With heating, water loss would expectedly increase the N/C ratio, while ammonia loss would decrease it. Samples before and after heating to 300 °C showed a net decrease in the N/C ratio, indicating that the second DSC endotherm and the TGA weight loss in zone 3 corresponds to decomposition. Decomposition of AUH releases more ammonia than water, due to the sub-stoichiometric water occupancy. Changes in the fingerprint region of FTIR spectra of heated and unheated material also support these assignments (Figure S4).

### A Better Model for Biogenic Uroliths

**Figure 2:**
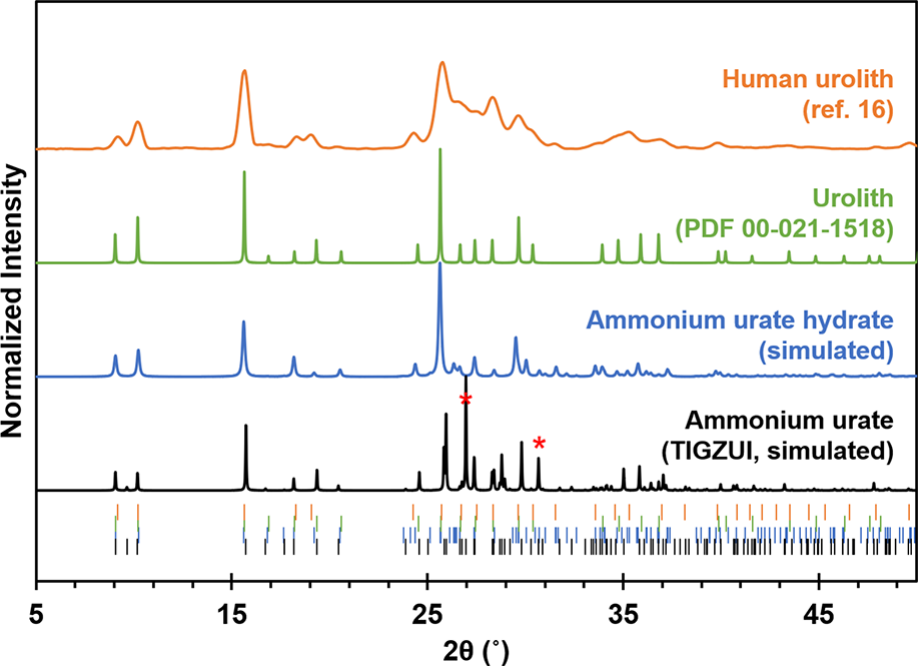
Comparison of PXRD patterns from two ammonium urate uroliths, and PXRD patterns simulated from the hydrate and anhydrate cif files. The two intense peaks labeled with a red asterisk are the ones most dissimilar to the biogenic sample. PXRD patterns are presented on a Cu scale (λ = 1.5406 Å).

Figure 2 compares the digitized PXRD pattern of the human urolith from Friedel et al.,^16^ a second ammonium urate urolith pattern retrieved from the PDF-4 database (PDF 00-021-1518),^22^ and the simulated PXRD patterns of triclinic AUH and monoclinic AU.^15^ The low angle peak positions at 2θ < 24° in all four patterns are remarkably similar, though differences become more apparent at higher angles. The most intense PXRD peak of the reported AU structure (2θ = 26.93°) is not particularly intense in either urolith. The sixth highest intensity peak in AU (2θ = 30.88°) is also not prominent in the uroliths. The PXRD patterns of natural samples can vary, as illustrated by the urolith data in orange and green (see also PXRD patterns in refs (23−26)). There are many potential explanations for this, including poor crystallinity, impurities, and disorder, as well as the likelihood that biogenic forms are often mixtures of AUH and AU (and potentially other forms). When accounting for both the peak positions and relative intensities, the AUH model is a much better match for the green urolith pattern, which we assume has a composition that is predominantly AUH. The relative intensities in the orange pattern suggest it is more likely a mixture of AUH and AU.

### Time-Resolved Synchrotron PXRD (sPXRD)

Motivated by Friedel et al.’s^16^ structural gradient hypothesis and suggestion that the source of the peak broadening in uroliths warranted additional investigation, we turned to time-resolved in situ sPXRD. Structural changes observed under temperature ramping conditions were key to revealing the unique aspects of AUH that provide a molecular-level explanation for the observed broadening and the variability in biogenic samples.

AUH was heated at 10 °C/min under a constant He flow (RH = 0%). With continuous data acquisition, this method enabled a high-resolution pattern to be obtained every ∼30 s. First, AUH was heated to 150 °C and then cooled back to ambient temperature. sPXRD patterns before and after heating to 150 °C showed essentially no changes in the peak positions and only small changes in the absolute intensities of some peaks (Figure S6). We considered the possibility that water loss from AUH yields an isomorphous dehydrated phase but concluded that was unlikely. The simulated PXRD of a hypothetical water-free isomorph indicates this would result in significant changes in the relative intensities of the low angle peaks (Figure S7).

**Figure 3:**
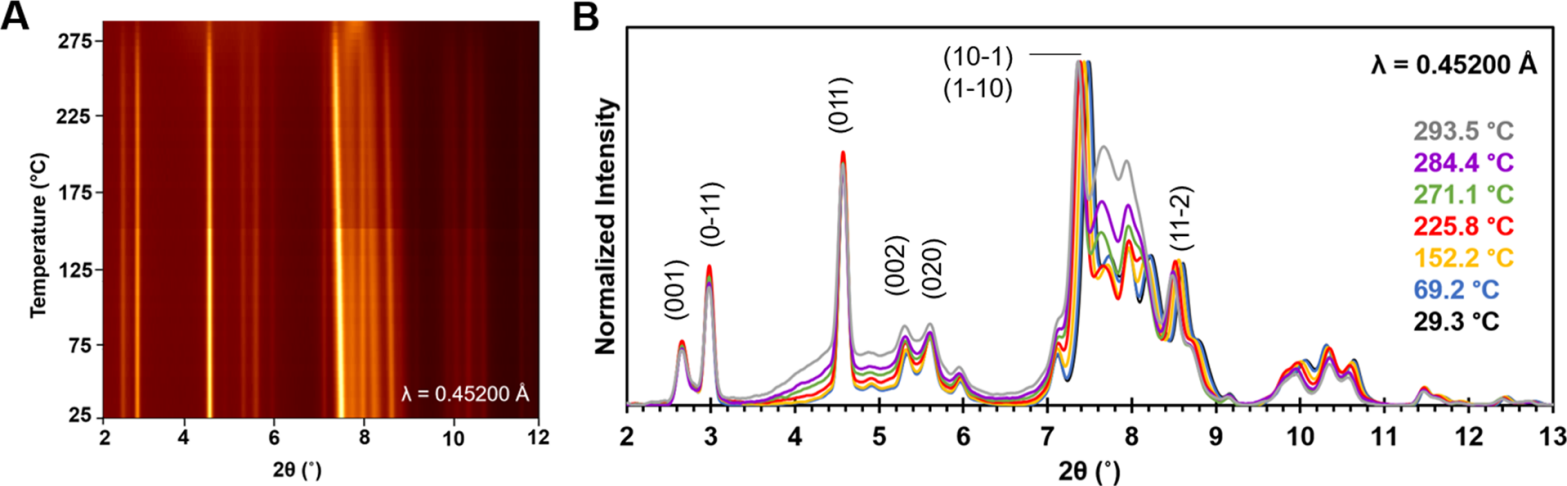
(A) Contour plot of synthetic AUH heated at 10 °C/min at 0% RH. (B) Sections of seven sPXRD patterns from the contour plot to illustrate structural changes in the material with temperature. Intensities are normalized to the highest intensity peak. The 2θ range is based on λ = 0.45200 Å.

A second sample of AUH from the same batch was heated to 300 °C to elucidate the structure changes that occur above 150 °C. The 62 patterns collected are presented as a contour plot in Figure 3A with select sPXRD patterns shown in Figure 3B (for detailed views, see also Figure S8). Above 150 °C, and especially above ∼230 °C, there are some noticeable changes in the diffractograms. Some of the peak positions shift owing to thermal expansion, though this is only seen in peaks that have an *a*-axis component, e.g., (10–1), (11–2). The (0*kl*) peaks such as (001), (011), and (020) do not appear to shift at all over this broad temperature range. This means that thermal expansion largely occurs between the π-stacked sheets rather than within them.

There is significant anisotropic broadening in the (011) peak, the plane which bisects the short side of the pores and parallels the head...head hydrogen-bonded urate dimers. With increased thermal motion at higher temperatures, the broadening suggests that the 2-D sheets are somewhat flexible. Interestingly, the (01–1) peak, which also bisects the sheets, does not exhibit broadening. This presumably reflects the comparatively stronger interactions in the direction of the continuous array of side...side hydrogen-bonded urates. Flexing of the 2-D sheets is likely a low-energy process, as it can be accommodated by modest changes in the intermolecular hydrogen bonding angles as is seen in other strongly hydrogen-bonded 2-D sheets.^27^

At temperatures above 150 °C, there is also a noticeable increase in the *relative* diffraction intensity between 2θ = 7.7−8.2° (corresponds to 26.2–27.9° on Cu scale). AUH does not contribute much intensity to peaks in this 2θ region; however, this is where the most intense peaks from AU are expected. This suggests the ratio of AUH:AU changes with temperature. Likely scenarios that could give rise to a changing phase mixture include: (1) AUH dehydrates to AU, (2) AUH becomes amorphous and/or decomposes starting at a lower temperature or proceeds at a comparatively faster rate than AU, or (3) some combination of these processes.

**Figure 4:**
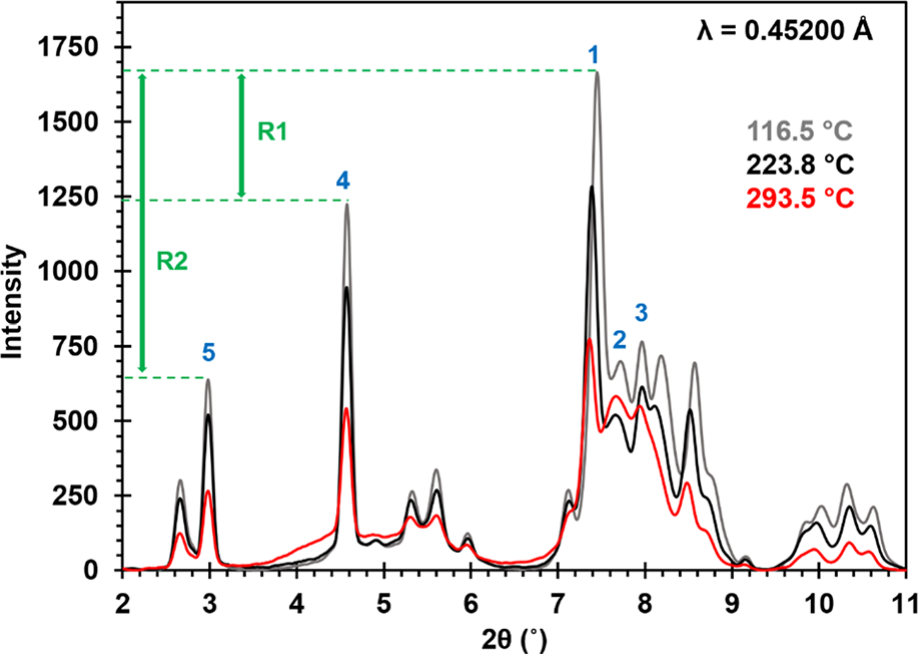
Overlay of sPXRD patterns collected at 116.5, 223.8, and 293.5 °C. AUH and AU contribute diffraction intensity to peaks 1, 2, and 3 (blue), though AUH makes a greater contribution to peak 1, while peaks 2 and 3 are primarily due to AU. R1 and R2 (green) refer to intensity ratios of *I*_(peak 1)_/*I*_(peak 4)_ and *I*_(peak 1)_/*I*_(peak 5)_, respectively. The 2θ range is based on λ = 0.45200 Å, and the *Y*-axis is measured intensity after background correction.

In an effort to distinguish between these options, the *absolute* intensities of select peaks were examined as a function of temperature (Figure S9). Such comparisons are useful for providing qualitative insight, though precise quantification of the ratios is complicated by thermal expansion and Debye–Waller effects, as well as the significant overlap in the peak positions. In Figure 4, sPXRD patterns at 116.5, 223.8, and 293.5 °C are overlaid with the *Y*-axis units in absolute counts. The peaks labeled 1, 2, and 3 have intensity contributions from both AUH and AU, though a much larger fraction of the peak 1 intensity comes from AUH, whereas a larger fraction of the peak 2 and 3 intensities derives from AU. Peaks 4 and 5 have roughly equal contributions from both forms. The absolute intensities of peaks 1, 4 and 5 decrease at the three temperatures, but the ratios of the intensities R1 = *I*_(peak 1)_/*I*_(peak 4)_ ∼ 1.4 and R2 = *I*_(peak 1)_/*I*_(peak 5)_ ∼ 2.6 are fairly consistent. This suggests that most of the intensity loss is due to decomposition of AUH, since conversion to AU would be expected to show a comparatively faster decline in peak 1. The absolute intensities of peaks 1, 2, and 3 were also compared. Between 116.5 and 223.8 °C, all three peaks show a decrease in intensity of ∼23 ± 3%. However, between 223.8 and 293.5 °C, peak 1 decreases by ∼40%, while changes in peaks 2 and 3 are comparatively smaller. This indicates AUH disappears at a rate faster than AU at temperatures >223.8 °C. The TGA weight loss observed in zone 3 (Figure 1C) is also consistent with partial decomposition in this higher temperature range.

### Comparison of AUH and AU Structures

**Figure 5:**
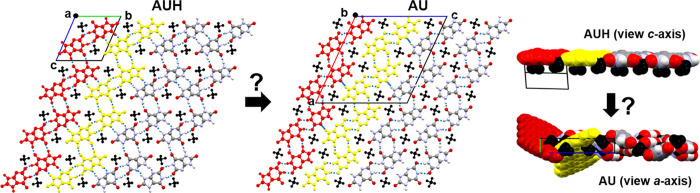
Two views of AUH and AU. Ammonium ions (black) and select urate ions (red and yellow) are colored. Water molecules in AUH are not shown. AUH is viewed normal to the 2-D hydrogen bonded plane and down the *c*-axis. AU is viewed normal to (010) and down the *a*-axis. Topotactic transformation from AUH to AU may be hypothetically possible but has not yet been experimentally demonstrated.

Side-by-side comparison of the AUH and AU structures shows that the two share a number of common structural elements. Top-down and side-on views of the 2-D sheet in AUH and the (010) AU plane are presented in Figure 5. Select urate ions are colored red and yellow for discussion purposes. The top-down views appear remarkably similar, though this perspective can be misleading as it is only in AUH that urate ions form planar 2-D hydrogen-bonded sheets. In AU, the red urates form an infinite hydrogen-bonded 1-D chain along the *a*-axis, as do the yellow urates, but any pair of adjacent red and yellow urate chains are connected by hydrogen bonds at only two points. The 1-D chains are twisted out of the (010) plane, such that each red chain is connected to many neighboring yellow chains on either side creating a 3-D hydrogen bonding network. The end-on views offer a better visualization for this twisting. The twisting enables a shorter repeat distance in the face-face π-stacks in AU (3.50 Å) compared to AUH (3.68 Å). The similarity in the packing motifs helps to explain the significant overlap in the low-angle peak positions.

Whether AUH and AU can interconvert remains an open question, though the data suggest it is not a dominant process under the rapid heating conditions explored here. Under other conditions (e.g., extended aging times) transformation between forms may still be possible. Calculation of the packing fraction of both AUH and AU in PLATON^28^ showed they are identical at 0.79. This is on the high end for molecular crystals.^29^ If an AUH to AU transformation were to occur under other conditions, topochemical arguments point to a preferred molecular-level trajectory. With increased thermal motion at higher temperatures and flexibility in the 2-D sheets, torque may eventually cause the planar sheets in AUH to buckle and fracture. By fracturing along (010), the red and yellow 1-D chains that were once part of the same 2-D sheet are no longer. However, each chain could re-establish hydrogen bonds with chains from other fractured sheets, and the shorter face-face π-stacks would offer an additional benefit. One could envision other scenarios where buckling and fracture of the 2-D planes along other directions could lead to different polymorphs.

## Conclusions

Ammonium urate stones have been a known medical issue for several centuries, though the development of an accurate structural model has remained elusive for much of that time owing to the unique challenges of this system. In this work, we put forth a model for AUH which is a better fit to the most intense peaks in the PXRD patterns of uroliths and assert that different ratios of AUH and AU account for much of the variability seen in biogenic samples. Flexibility in the 2-D planar hydrogen-bonded urate framework also provides an explanation for some of the unusual features seen in the PXRD patterns. We are indebted to previous authors who pursued structural studies on this material without the benefit of advanced instrumentation or an existing structure model. Given the rapid crystallization of ammonium urate, the fact that it generates at least two forms with similar densities, and the potential for disorder and/or polytypism in this system, it would not be surprising if additional ammonium urate forms were identified at some future date.

## Experimental Section

### AUH Synthesis

Anhydrous uric acid (90 mg, Aldrich 99 + %) was immersed in a 25% ammonium hydroxide solution (30 mL, Acros Organics) and maintained at room temperature. This molar ratio of uric acid:ammonia is ∼800:1. The resulting sample was typically isolated by vacuum filtration after 24 h and hand-ground in a mortar and pestle before analysis. Most reactions yielded a mixture of AUH, some amorphous material, and varying amounts of the anhydrous ammonium urate (AU) previously reported. We attempted to optimize the fraction of AUH by varying the reaction parameters, e.g., number of days in solution and lowering the reaction temperature but saw no obvious correlation with the final composition.

### Scanning Electron Microscopy (SEM)

Crystal samples were attached to 12 mm aluminum SEM mounting stubs with conductive carbon tape. SEM images were obtained on a Zeiss Supra 55-VP scanning electron microscope operated under vacuum at 1 kV accelerating voltage using secondary electron mode detection.

### Thermal Analysis

DSC data were collected on a TA Instruments Discovery DSC 25 using 3–5 mg sample and capped but unsealed aluminum pans. Samples were heated at 10 °C/min from room temperature to 400 °C.

TGA weight loss was measured using a TA Instruments SDT_Q600. Samples (2–5 mg) were heated in open ceramic pans under nitrogen flow (50 mL/min) at 10 °C/min to a maximum temperature of 300 °C. The reported weight loss is an average of triplicate measurements. The calculated water content of AUH with monohydrate stoichiometry is 8.86%. With 0.73 water occupancy, water content = 6.47%.

### Elemental Analysis

Elemental combustion analysis was performed using a PerkinElmer 2400 CHN Series II Elemental Analyzer with acetanilide as a calibration standard. All samples were run in triplicate.

### Spectroscopy

Raman spectra were collected using a Horiba LabRAM HR Evolution confocal microscope with a 532 nm (100 mW) laser. Data are 5 to 20 accumulations of 10 to 30 s scans. Spectra were background subtracted to remove fluorescence and intensity spikes from changes in the detector range. Fourier-transform infrared (FT-IR) spectra were recorded on a PerkinElmer Spectrum-Two FT-IR spectrophotometer equipped with a UATR-TWO diamond ATR attachment. Each spectrum represents an average of 10 scans.

### Powder X-ray Diffraction

PXRD data were collected on ground samples using a Bruker D2 Phaser 2nd generation X-ray diffractometer (Cu *K*α radiation, reflection mode, 300 W, 0.02° step, 0.5 s steps, rotate at 15 rpm, 2θ range = 8–70° or 8–90°, zero background Si disk) or a Bruker Apex DUO X-ray diffractometer (Cu *K*α radiation, transmission mode, 50 kV, 30 mA, 2θ range = 7–60°, Kapton capillary). PXRD patterns were compared against uric acid and ammonium urate structures in the Cambridge Structural Database (CSD),^30^ the 2023 releases of the ICDD’s PDF-4+ database,^31^ and the PDF-4/Organics database.^32^

### Synchrotron PXRD (sPXRD)

Variable-temperature sPXRD data were collected at the Advanced Photon Source (APS) beamline 17-BM-B with a λ = 0.45200 Å (27.4302 keV). Manually ground samples were loaded in Kapton capillaries (Cole-Palmer 95820-07, OD: 1.0 mm, ID: 0.9 mm), stoppered with quartz fiber filters, placed in a flow cell,^33^ and rocked 15° (at ∼2–3° per minute) while under a constant He gas flow (5 mL/min). With a beam size = 300 μm, detector distance = 600 mm, and a 1 s per image exposure time (summed over 10 images), sPXRD patterns were collected every ∼30 s while heating at 10 °C/min. GSAS-II^34^ was used for image processing and integration.

### Rietveld Refinements

Refinements were carried out using the whole pattern fitting (WPF) module in JADE Pro. With samples that have small crystallite sizes and were often phase mixtures, the diffraction patterns showed significant peak overlap wherein the number of observed experimental peaks exceeded the number of variables. Our block refinement strategy followed general Rietveld procedures for multi-phase samples using a procedure previously applied to cements.^35^ Typically unit cells, scale factors and an overall temperature factor are initially refined, the refinement results are analyzed, and then peak profiles are refined in later cycles. JADE Pro does not allow for the refinement of hydrogen atom positions. Atomic parameters and site occupancies were selectively refined in the final steps.

An initial energy-minimized structure for ammonium urate hydrate was calculated using VASP^21^ and DFT based on molecular fragments from refcode: HOZSUL.^16^ The structure was later modified to replace the ammonia molecule with water based on elemental analysis data. Initial refinements matched the main features in experimental PXRD patterns but did not fit exactly until the water molecule was allowed to refine its site occupancy and position. In later refinement cycles where all atoms were allowed to move, the C–O(11) and O–H water bonds were longer than expected. The position of O(11) was reset, and during refinement, the water oxygen moved creating a more appropriate bond geometry. The water occupancy repeatedly converged at 0.73 in different data sets. Ammonium urate was modeled using refcode: TIGZUI.

Refinements took advantage of some special features in JADE Pro. First, different backgrounds were required depending on the diffraction data source. Data collected at the APS and Georgetown University (GU) used transmission optics and Kapton capillaries. Data collected at the ICDD used reflection geometry and a zero background holder (polished off-cut silicon wafer). A refinable 5th order polynomial was used to eliminate the Kapton contributions in GU and APS data sets, while a simpler 3rd order polynomial was used with ICDD data sets to account for the angularly dependent air scatter. JADE Pro also allows the user to put in an amorphous pattern contribution and offers a variety of profile fitting functions, including several used for asymmetric peaks profiles influenced by various structural defects in poorly crystalline materials.

Multiple data sets refined to the same AUH unit cell, all with *R*_wp_ between 3.5−8.5% (Table S2). CCDC deposition number: 2278848.

## Supporting Information Available

SEM images, crystallographic data, DSC thermograms, FTIR and Raman spectra, PXRD, and sPXRD and data tables (PDF)
