# Supplementary material for: An Improved Model for Biogenic Ammonium Urate
Source: Cryst Growth Des. 2023 Aug 16;23(9):6953–9. doi: 10.1021/acs.cgd.3c00789 (PMC10486279; doi:10.1021/acs.cgd.3c00789)
Supplement: Supplementary file 1 — cg3c00789_si_001.pdf [file cg3c00789_si_001.pdf]

Supplementary Information

An Improved Model for Biogenic Ammonium Urate

Alyssa M. Thornton[1], Timothy G. Fawcett,[2*] James A. Kaduk,[3,4] YuJai Lin,[1] and Jennifer A. Swift[1*]

[1] Department of Chemistry, Georgetown University, Washington, DC 20057 USA

[2] International Centre for Diffraction Data, Newtown Square, PA 19073 USA

[3] Illinois Institute of Technology, 3101 S. Dearborn St., Chicago, IL 60616, USA

[4] USA North Central College, 131 S. Loomis St., Naperville, IL 60540, USA

*corresponding author email: jas2@georgetown.edu

Table of Contents

	page
**Figure S1.** SEM image of uric acid before and after reaction in 800-fold excess ammonium hydroxide.	3
**Table S1.** Crystallographic data for reported ammonium urate phases.	3
**Table S2.** Block refinements of replicate syntheses.	4
**Figure S2.** Rietveld refinement profiles of synthetic products. (top) Refinement of data set 1 collected on a Bruker Apex DUO diffractometer (Rwp = 3.45%). (bottom) Refinement of data set 4 collected at 17-BM-B (Rwp = 5.55%). The difference plots for each are shown at the top in red.	5
**Figure S3.** Overlaid DSC thermograms of synthesized AUH heated to 400 °C at 10 °C/min. The red trace was collected the same day it was harvested from solution. The black trace is material that was exposed to ambient air for four days before DSC analysis.	6
**Table S3.** Calculated C, H and N content in different hypothetical ammonium urate phases, and measured C, H, and N values in synthetic material.	6
**Figure S4.** (top) FT-IR spectra of AUH as synthesized, after heating to 150°C, and after heating to 300°C. The FT-IR spectrum of uric acid powder is included for reference. (bottom) Unheated and heated uric acid powder are identical. All spectra are collected at room temperature.	7
**Figure S5.** Raman data of uric acid (black) and the synthesized AUH (red).	8
**Figure S6.** Overlay of room temperature sPXRD patterns before and after heating the synthetic material to 150 °C. The 2θ scale corresponds to λ = 0.45200 Å.	8
**Figure S7.** Comparison of simulated PXRD patterns of AUH with water occupancies of 1 and 0. Calculated packing fractions (PF) are indicated.	9
**Figure S8.** Expansion of the regions between 2θ = 2.0 – 6.5° and 6.5 – 9.5°. The 2θ scale corresponds to λ = 0.45200 Å.	10
**Figure S9.** sPXRD data shown on an absolute intensity scale. The 2θ scale corresponds to a λ = 0.45200 Å.	10
**Table S3.** AUH Calculated lattice parameters and fractional atom coordinates.	11

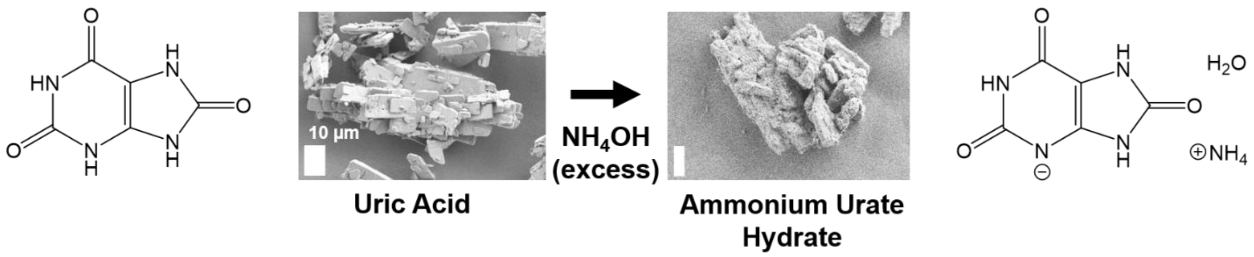

Figure S1. SEM image of uric acid before and after reaction in 800-fold excess ammonium hydroxide.

Table S1. Crystallographic data for reported ammonium urate phases.

	Tettenhorst and Gerkin[1]<br><br>PXRD	Rimer et al. (TIGZUI)[2]<br><br>3D EM	Rimer et al. (TIGZUI01)[2]<br><br>PXRD	Friedel et al. (HOZSUL)[3]<br><br>PXRD	Ammonium urate hydrate (AUH)<br><br>PXRD
	$C_5H_3N_4O_3 \cdot NH_4$	$C_5H_3N_4O_3 \cdot NH_4$	$C_5H_3N_4O_3 \cdot NH_4$	$C_5H_3N_4O_3 \cdot NH_4 \cdot NH_3$	$C_5H_3N_4O_3 \cdot NH_4 \cdot 0.73 H_2O$
	monoclinic	C2/c	C2/c	P-1	P-1
a (Å)	17.356	21.385	21.3801	3.650	3.6863
b (Å)	3.528	3.5300	3.5000	10.215	10.0727
c (Å)	11.282	20.080	20.0802	10.597	10.6368
α (°)	90	90	90	113.9	113.46
β (°)	94.23	114.10	114.10	91.1	90.53
γ (°)	90	90	90	92.3	91.65
V (Å^3)	689.11	1383.7	1371.63	361	362.07
Z	4	8	8	2	2
V/Z	172.27	172.96	171.45	180.5	181.03

1. Tettenhorst, R. T.; Gerkin, R. E., *Powder Diffr.* **1999,** *14* (4), 305-307.
2. Tang, W.; Yang, T.; Morales-Rivera, C. A.; Geng, X.; Srirambhatla, V. K.; Kang, X.; Chauhan, V. P.; Hong, S.; Tu, Q.; Florence, A. J.; Mo, H.; Calderon, H. A.; Kisielowski, C.; Hernandez, F. C. R.; Zou, X.; Mpourmpakis, G.; Rimer, J. D., *Nature Communications* **2023,** *14* (1), 561.
3. Friedel, P.; Bergmann, J.; Kleeberg, R.; Schubert, G., *Z. Kristallogr. Suppl.* **2006,** *23*, 517-22.

Table S2. Block refinements of replicate syntheses.

	**1**	**2**	**3**	**4**
Composition	AUH: 47%<br>AU: 43%<br>Amorphous: 10%	AUH: 51%<br>AU: 46%<br>Amorphous: 3%	AUH: 63%<br>AU: 35%<br>Amorphous: 2%	AUH: 48%<br>AU: 47%<br>Amorphous: 5%
Approx. counts	12,000	100,000	170,000	1000
Data collection	GU	ICDD	ICDD	APS
Rwp	3.45%	7.92%	8.45%	5.55%
notes		In growth solution 1 day	In growth solution 4 days	In growth solution 1 day

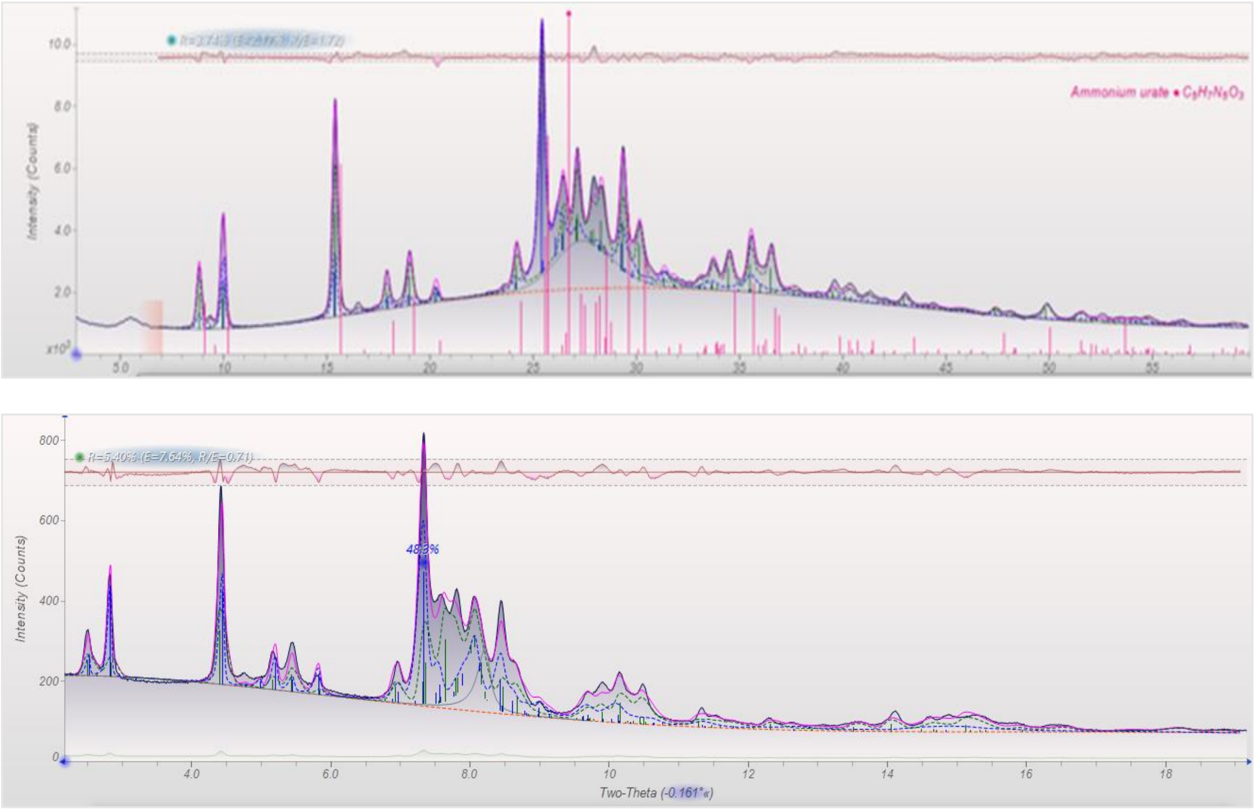

Figure S2. Rietveld refinement profiles of synthetic products. (top) Refinement of data set 1 collected on a Bruker Apex DUO diffractometer (Rwp = 3.45%). (bottom) Refinement of data set 4 collected at 17-BM-B (Rwp = 5.55%). The difference plots for each are shown at the top in red.

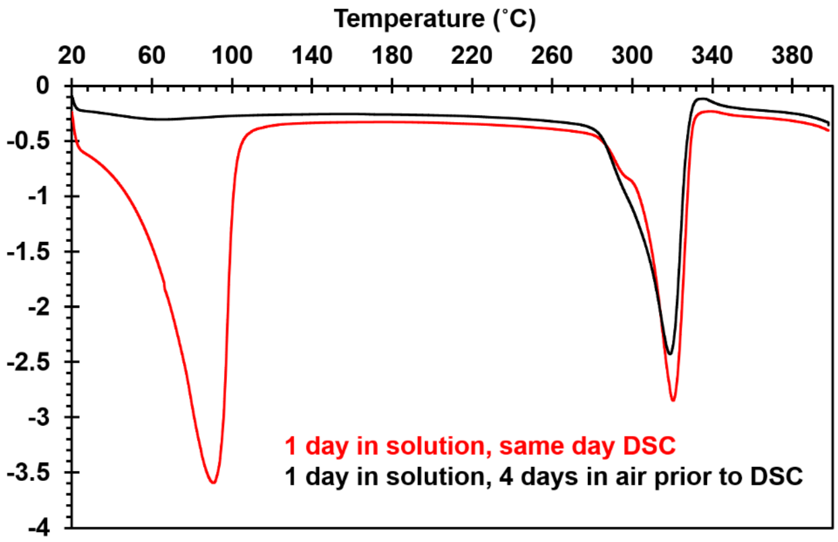

Figure S3. Overlaid DSC thermograms of synthesized AUH heated to 400 °C at 10 °C/min. The red trace was collected the same day it was harvested from solution. The black trace is material that was exposed to ambient air for four days before DSC analysis.

Table S3. Calculated C, H and N content in different hypothetical ammonium urate phases, and measured C, H, and N values in synthetic material.

	C	H	N
$C_5H_3N_4O_3{\cdot}NH_4$ (MW = 185)	32.43%	3.78%	37.83%
$C_5H_3N_4O_3{\cdot}NH_4{\cdot}NH_3$ (MW = 202)	29.70%	4.95%	41.58%
$C_5H_3N_4O_3{\cdot}NH_4{\cdot}H_2O$ (MW = 203)	29.56%	4.43%	34.48%
$C_5H_4N_4O_3$ (MW = 168)	35.71%	2.38%	33.33%
Expt. (before heating)	31.68 ± 0.02%	3.57 ± 0.06%	36.06 ± 0.09%
Expt. (heated 300°C)	33.35 ± 0.03%	3.27 ± 0.03%	35.3 ± 0.2%

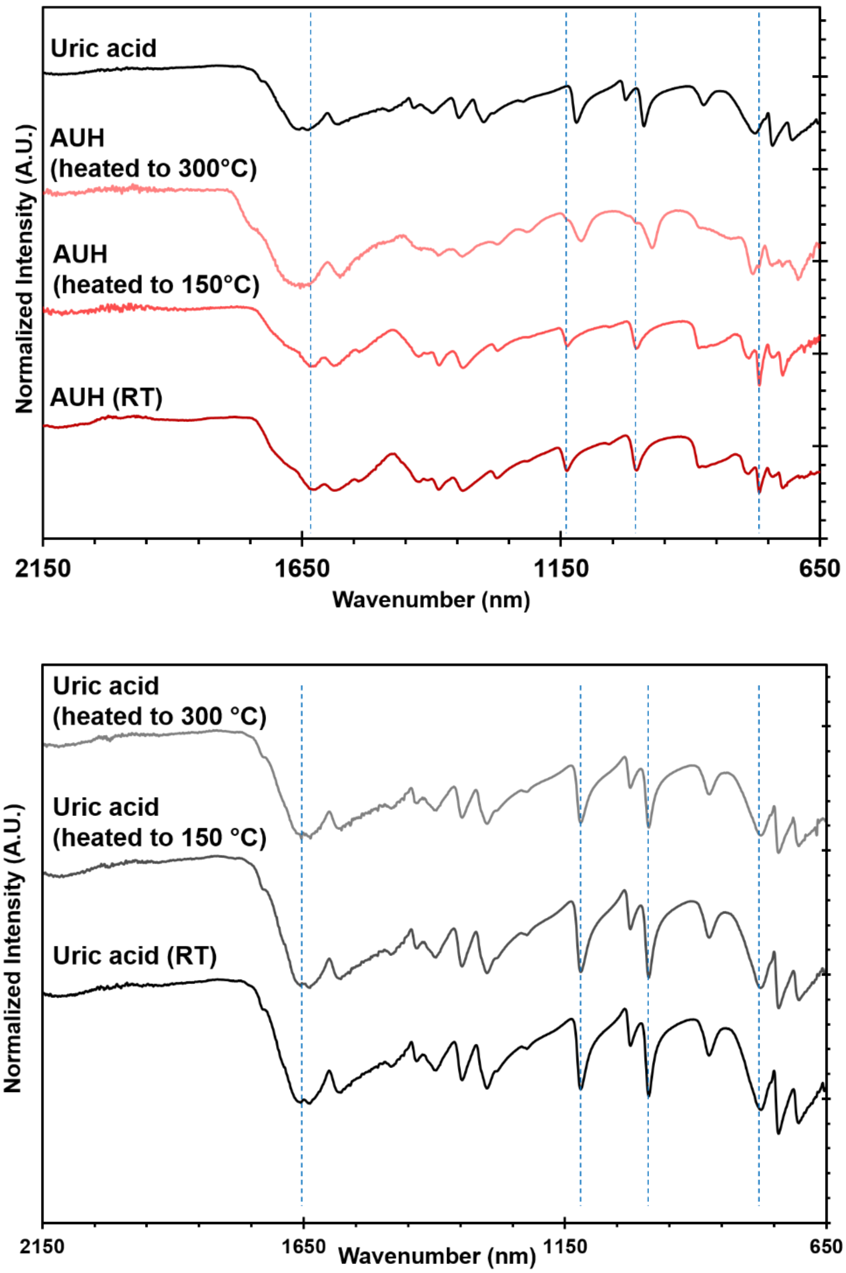

Figure S4. (top) FT-IR spectra of AUH as synthesized, after heating to 150°C, and after heating to 300°C. The FT-IR spectrum of uric acid powder is included for reference. (bottom) Unheated and heated uric acid powder are identical. All spectra are collected at room temperature.

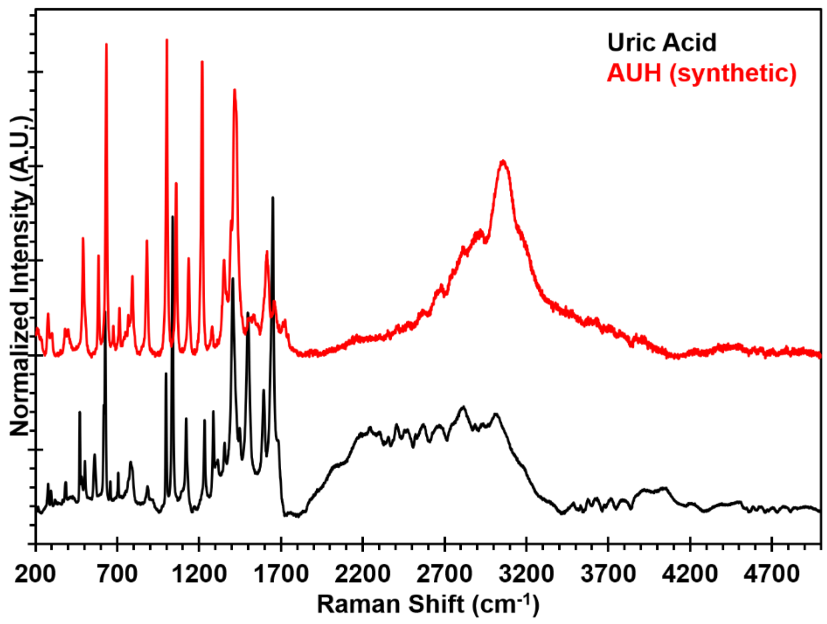

Figure S5. Raman data of uric acid (black) and the synthesized AUH (red).

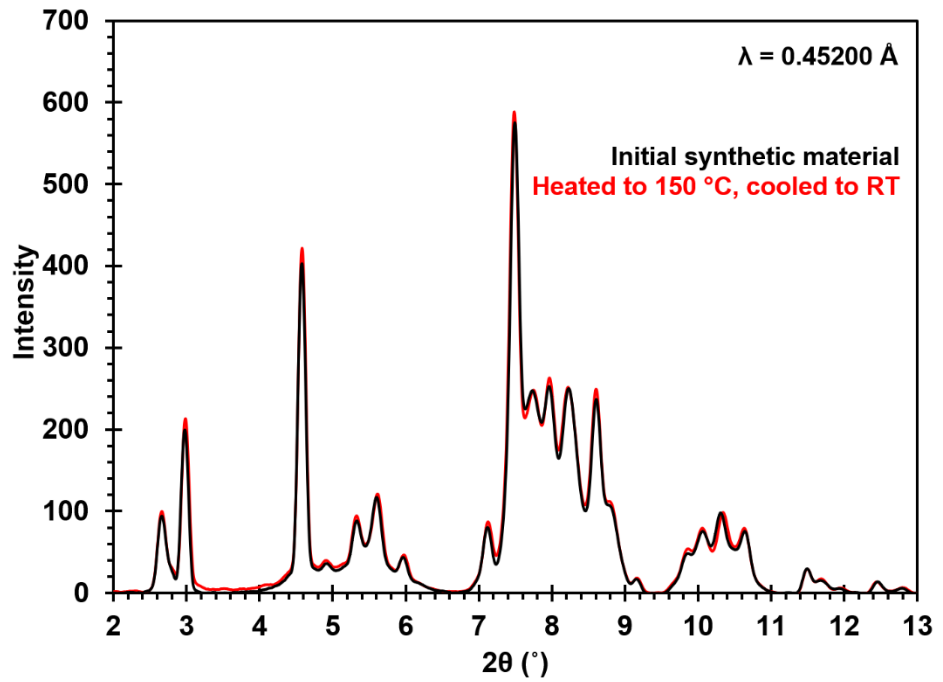

Figure S6. Overlay of room temperature sPXRD patterns before and after heating the synthetic material to 150 °C. The 2θ scale corresponds to λ = 0.45200 Å.

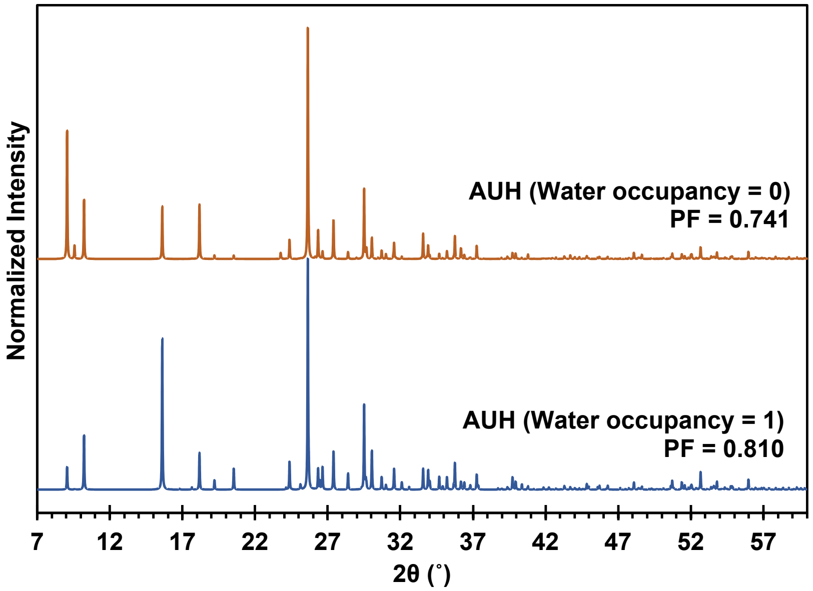

Figure S7. Comparison of simulated PXRD patterns of AUH with water occupancies of 1 and 0. Calculated packing fractions (PF) are indicated.

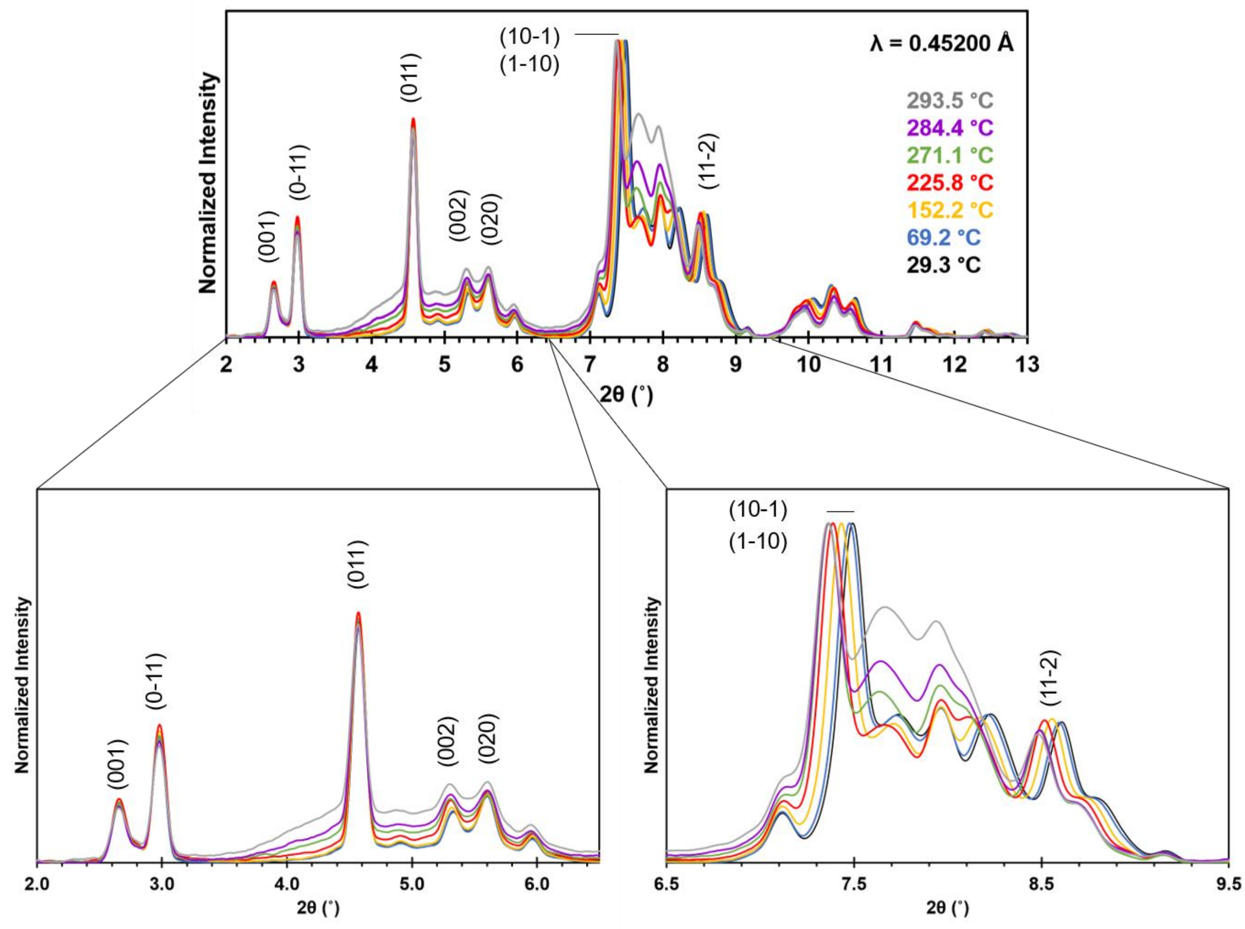

Figure S8. Expansion of the regions between 2θ = 2.0 – 6.5° and 6.5 – 9.5°. The 2θ scale corresponds to λ = 0.45200 Å.

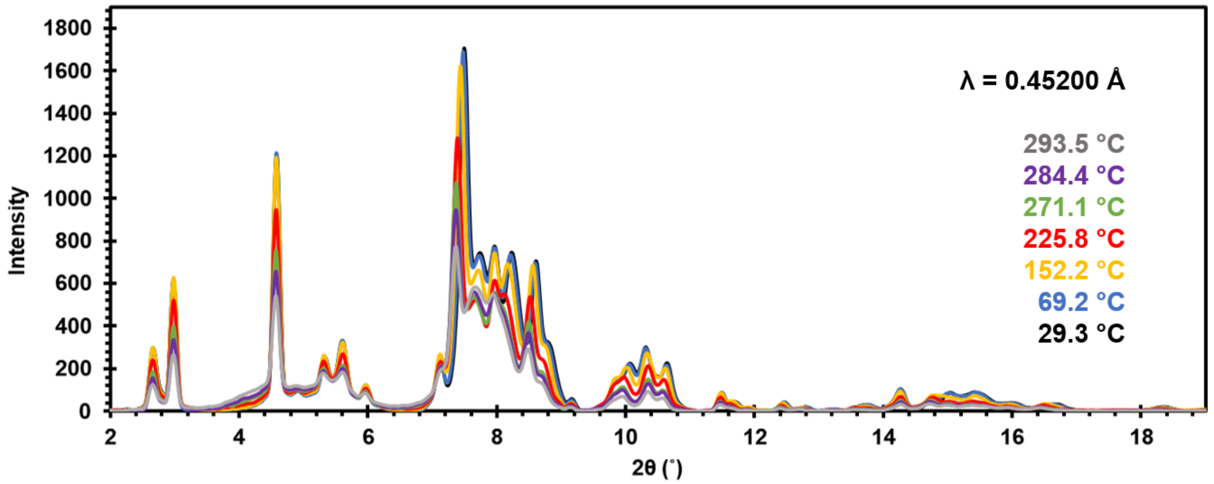

Figure S9. sPXRD data shown on an absolute intensity scale. The 2θ scale corresponds to a λ = 0.45200 Å.

Table S3. AUH Calculated lattice parameters and fractional atom coordinates.

Triclinic

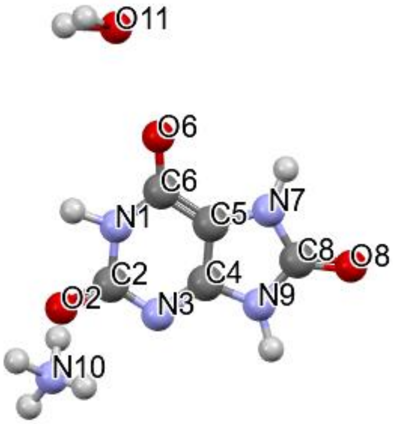

Space group:	P-1
a	3.68633
b	10.07274
c	10.63675
alpha	113.4611
beta	90.52695
gamma	91.64735
cell volume	362.07
Z	2.0

Fractional coordinates x, y, z (occupancy)

C6	0.22706	0.55521	0.22931	
N1	0.40968	0.56189	0.34322	
C2	0.55372	0.67439	0.45904	
N3	0.51693	0.80512	0.46688	
C4	0.35385	0.80995	0.35512	
C5	0.21886	0.69538	0.23633	
N9	0.29685	0.93331	0.33874	
C8	0.1411	0.89777	0.21098	
O8	0.09247	0.98965	0.16052	
N7	0.07886	0.75295	0.14837	
O6	0.0859	0.43758	0.13125	
O2	0.69891	0.6511	0.55842	
H14	0.98134	0.69362	0.04829	
H13	0.37094	0.46473	0.37035	
H15	0.3661	0.04256	0.41129	
N10	0.19349	0.76465	0.76857	
H17	0.04686	0.6746	0.79369	
H18	0.29856	0.69781	0.66776	
H19	0.40669	0.79358	0.83859	
H16	0.02714	0.82762	0.76396	
O11	0.41800	0.12800	-0.06600	(0.733)
H20	0.59383	0.08417	0.96408	(0.733)
H21	0.17787	0.08984	0.96376	(0.733)
